# Supplementary material for: Defining a Treatment Model for Self-Management of Fatigue in Rehabilitation of Acquired Brain Injury Using the Rehabilitation Treatment Specification System
Source: J Clin Med. 2023 Apr 28;12(9):3192. doi: 10.3390/jcm12093192 (PMC10179474; doi:10.3390/jcm12093192)
Supplement: Supplementary file 1 [file jcm-12-03192-s001.zip › jcm-2253659-supplementary.pdf]

SUPPLEMENTARY MATERIALS

Table S1. Characteristics of service providers and researchers.

Characteristic	Service providers									Researchers	
	A.T.	K.N.	B.B.	T.K.	M.T.	M.S.	H.E.	H.J.	P.S.	F.D.	T.S.
Profession	OT	NP	PT	OT	NP	NP	OT	OT	PT	NP	OT
Sex	Female	Female	Female	Female	Female	Female	Female	Female	Female	Male	Female
Case assignment											
A	X	X	-	-	-	-	-	-	-	-	-
B	X	X	X	-	-	-	-	-	-	-	-
C	-	-	-	X	-	-	-	-	-	-	-
D	-	-	-	X	X	X	-	-	-	-	-
Interviewee	X	-	-	X	-	-	-	-	-	-	-
Workshops	-	X	X	-	-	-	X	X	X	X	X

Note: OT = Occupational Therapist; NP = Neuropsychologist; PT = Physiotherapist.

Table S2. Standardized outcomes pre- and post-rehabilitation.

Variable (range)	Case A		Case B		Case C		Case D		Mean (SD)	
	Pre	Post	Pre	Post	Pre	Post	Pre	Post	Pre	Post
DMFS										
IF (11–55)	28	36	48	42	35	43	31	22 *	35.5 (8.8)	35.8 (9.7)
SC (9–45)	29	25	31	25	26	26	30 *	23	29.0 (2.2)	24.8 (1.3)
MF (7–35)	23	21	33	30	27	30	26	12	27.2 (4.2)	23.2 (8.6)
PF (6–30)	14	15	19	19	21	23	20	17 *	18.5 (3.1)	18.5 (3.4)
CF (5–25)	11	11	14	15	16	19	18	17	14.8 (3.0)	15.5 (3.4)
DASS-21										
Depression (0–42)	2	0	18	14	2	2 *	4	2	6.5 (7.7)	4.5 (6.4)
Anxiety (0–42)	4	4	28	20	0	0	2	0	8.5 (13.1)	6.0 (9.5)
Stress (0–42)	4	4	18	20	2	2	2	0	6.5 (7.7)	6.5 (9.2)
EQ-5D-5L										
Index (-0.624–1.000)	0.859	-	0.727	0.626	0.783	0.719	0.742	0.799	.778 (.059)	-
PSQI Global (0–21)	8	6	6	6	7	6	7	6	7.0 (0.8)	6.0 (0.0)
GSE total (10–40)	33	27	18	23	28	19	28	28 *	26.8 (6.3)	24.2 (4.1)
RRTW scale										
RRTW-PC (3–15)	6	15	6	9	9	-	4	3 *	6.3 (2.1)	-
RRTW-C (3–15)	9	3	12	10	10	-	9	8	10.0 (1.4)	-
RRTW-E (4–20)	18	4	12	7	10	-	9	14	12.2 (4.0)	-
RRTW-B (3–15)	12	3	12	10	9	-	13	9	11.5 (1.7)	-

Note: DMFS = Dutch Multifactor Fatigue Scale (greater score indicates worse problems); IF = Impact of Fatigue; SC = Signs and Direct Consequences of Fatigue; MF = Mental Fatigue; PF = Physical Fatigue; CF = Coping with Fatigue; DASS-21 = 21-item Depression Anxiety Stress Scales (greater score indicates more symptoms); EQ-5D-5L = 5 level EQ-5D (greater score indicates better quality of life); PSQI = Pittsburgh Sleep Quality Index (greater score indicates worse sleep quality); GSE = Global Self-Efficacy scale (greater score indicates higher self-efficacy); RRTW = Readiness for Return to Work (greater score indicates more engagement in stage-related behavior); PC = Precontemplation; P = Contemplation; E = Prepared for action – self-evaluative; B = Prepared for action – behavioral. * One missing item score was imputed by the item mean.
